# “It’s research, our input can grow”: identifying health research priorities with Aboriginal and Torres Strait Islander communities—study protocol

**DOI:** 10.1186/s40900-023-00467-w

**Published:** 2023-07-28

**Authors:** Luciana Massi, Loretta Weatherall, Christine Nielsen, Maree Toombs, Bronwyn Fredericks, Kym M. Rae

**Affiliations:** 1 Mater Research Institute, University of Queensland, Level 3, Aubigny Place, QLD South Brisbane, Australia; 2 Sydney School of Public Health, University of Sydney, Camperdown, NSW Australia; 3 Office of the Pro-Vice Chancellor Indigenous Engagement, University of Queensland, St Lucia, QLD Australia

**Keywords:** Health research, Perinatal, Family health, Community involvement, Participatory action research, Aboriginal and Torres Strait Islander, Indigenous, Social justice, Equity, Yarning

## Abstract

**Background:**

In Australia, Aboriginal and Torres Strait Islander (Indigenous) families have strong, cohesive, and nurturing cultural practices that contribute to effective family functioning and child rearing. These practices can lead to positive effects on children and communities, and include kinship relations, traditional knowledge systems, collective community focus, respect for Elders contributions, and spirituality. However, poor health and wellbeing outcomes exist across the lifespan for Indigenous Australians. Health programs, services and research that support Indigenous women, babies and their families are a critical investment to improve birthing and health outcomes and impact the life trajectories of Indigenous Australians.

**Aim:**

The Indigenous Health Research Priorities study aims to identify the research priorities for families during the perinatal and early childhood period through a co-designed and collaborative process. This has been led by communities to determine the priorities identified with and for local Indigenous families in Queensland. This paper aims to report on engagement and involvement with Indigenous communities to identity health research priorities for families and presents preliminary findings of the research process including participants’ demographic information and feedback on the yarning sessions, as part of the study protocol.

**Methods:**

The study protocol showcases the Participatory Action Research approach, yarning sessions with clients and staff of three community-controlled health services to date, and Delphi workshop methods to prioritise the health issues identified during the yarns with corresponding communities. The study will undertake qualitative data collection and analysis to identify and report on community and health service research priorities for Indigenous families in Queensland. A short survey was conducted to collect participants’ demographic information. A feedback form with five open-ended questions was also administered to collect data on participants’ views and satisfaction with the research process.

**Preliminary results:**

This protocol paper reports on the participant demographic information and feedback on the research process and reactions to participating in the yarning sessions. There have been 12 yarning sessions in Far North Queensland to date. The qualitative analysis of these will be reported on in future, with South East Queensland and further sites to follow. Feedback from 61 community members and health professionals has highlighted they valued sharing stories, being heard, and feeling hopeful. Preliminary findings will be reported.

**Discussion:**

Identification of health research priorities will allow each organisation and region of Queensland to develop research initiatives and the translational outcomes that are a focus for their community members.

## Abstract (plain language summary)

Health programs and services designed to support Aboriginal and Torres Strait Islander (herein Indigenous) women, babies and their families are a critical investment to improve birthing and health outcomes, and potentially impact the life course of Indigenous Australians. The Indigenous Health Research Priorities study aims to identify research priorities for families during pregnancy, birthing, and early childhood through a collaborative consultation process. We engaged with community members, both clients and health care staff of three community-controlled health services in Far North Queensland. Yarning sessions were held to identify health research priorities with and for local Indigenous families. Feedback forms were collected to gauge engagement and satisfaction with the research process. Twelve yarning sessions with 61 participants highlighted they valued sharing stories, being heard, having a voice, and feeling hopeful. Identifying health research priorities will allow each organisation and region of Queensland to develop health programs and services and research initiatives that are important for their community members.

Once the yarning group data is analysed, we will return to discuss, prioritise, and reach consensus on those health issues identified during the yarns with communities, using a Delphi study. The Delphi will run as an interactive workshop using playing cards and group discussions, where participants will rank the importance of the health issues for their community. Prioritising the top 10 health issues will help to ensure research is designed better for and with communities, so that future research directions meet the needs identified and self-determined by Indigenous communities.

## Background

Globally, the First Peoples of many nations have long known that past generations influence the ongoing life experiences of those currently living. In this way, the Developmental Origins of Health and Disease (DOHaD) shows that the health of our parents and grandparents can directly impact on our own [29]. For many First People populations around the world, the DOHaD forms part of their worldview, traditional knowledge, and cultural practices. Many First People community members are mindful from their teachings that their own actions will affect those in future generations [37]. In Australia, Aboriginal and Torres Strait Islander (herein respectfully Indigenous) families have strong, cohesive, and nurturing cultural practices that contribute to effective family functioning and child rearing [1, 27, 34]. These practices can have positive effects on children and communities, and include kinship relations, traditional knowledge systems, a collective community focus with respect for Elders contributions, and spirituality [34].

However, the life expectancy of Australian Indigenous people is at least ten years lower than non-Indigenous Australians [21, 22]. This has resulted from significant health disparities, including three times greater maternal mortality and almost double the rates of infant mortality, higher rates of low birth weight and child hospitalisation when compared to other Australians [10, 22]. While there has been increasing policy attention, there has been little improvement in health and wellbeing outcomes for Indigenous peoples over many decades [9, 10, 22, 24, 26]. This can be attributed to ongoing negative impacts of colonisation and systemic racism, meaning many Indigenous families may require additional support to overcome structural and socio- economic determinants which perpetuate inequalities and adversely affect the health and well-being of families and communities [1, 20]. Health programs and services that support Indigenous women, babies and their families are a critical investment to improve birthing and health outcomes and to potentially impact the life trajectories of Australia’s Indigenous peoples [15, 22, 40].

The importance of interventions and support during the first 2,000 days—from conception until five years of age – are well evidenced as a crucial time for laying the foundations for nurturing healthy, thriving children into productive adulthood [40, 41, 46, 47]. Accordingly, many policies and services supporting Indigenous families focus on interventions during pregnancy and early life stages, also known as the perinatal period [22, 26]. The perinatal period encompasses pregnancy through to the end of the first year postpartum [2]. Research in perinatal and into the early childhood period (referred to in this paper as the period from infancy to the first five years) for families is an important time of life to focus on as it can potentially identify maternal health risks or negative health outcomes from the outset of a child’s life [2, 36].

The ‘Growing Deadly Families’ Aboriginal and Torres Strait Islander Maternity Services Strategy 2019–2025 written in partnership with the Queensland Aboriginal and Islander Health Council (QAIHC) states ‘nothing is more important than ensuring our future generations have the best start to life’ and have called for Aboriginal and Torres Strait Islander people to be equal partners in ‘decision-making, planning, delivery and governance’ (Queensland [48], p. 21). Leading Indigenous researchers have identified that national research priorities for Indigenous communities need to address health inequities and include strengthening and capacity building of Indigenous researchers, and focus on the health system and social and cultural determinants of health [25], National Health and Medical Research Council et al., 2018). They have highlighted that research that looks at impacts across the life course is critical in understanding the health trajectory of Indigenous peoples. However, it is imperative that research is led by or co-steered with Indigenous communities to identify local research priorities [28]. The National Agreement on Closing the Gap highlights that Indigenous people must be given every opportunity to genuinely self-determine research, and the design and delivery of services that affect them, and as result, better life and health outcomes are achieved for communities [22].

There is often mistrust of research, government organisations and their policies due to events that occurred within the living history of many Indigenous community members. Thus, partnered, and respectful relationships are needed to build sustainable research programs. Participatory Action Research (PAR) is an appropriate research method to use when conducting research with communities, and is “based on reflection, data collection, and action that aims to improve health and reduce health inequities through involving the people who, in turn, take actions to improve their own health” [12], pp., p.854). More specifically Community Based Participatory Action Research (CBPAR) principles aim to ensure there is authentic involvement and sharing in the way research is conceptualised, practiced and implemented, and not just the mere participation in collecting data [39], and provides an opportunity for reciprocity, or a reciprocal exchange to ensure community wellbeing [35, 54]. This importantly points to a socially just process of joint ownership between researchers and community members in the research process and the solutions identified, towards decolonised research [51]. McTaggart [39] suggests that PAR should contribute to improvements in the “understanding, practice and social situations for participants and others involved in the situation described” (p. 169), suggesting the practice will ultimately improve participants’ lives.

Likewise, the use of Delphi consensus techniques are appropriate methods for conducting PAR and have been previously used in Indigenous health research studies [30, 52, 53]. The Delphi technique involves guiding group opinion towards a final decision to answer questions through triangulation of group generated ideas, analytical techniques and the experience of the researcher [18]. The Delphi is a multistage technique, with each stage building on the previous one, and aims to encourage respondents to think through the complexity of the problem and produce specific, high quality ideas [18]. The Delphi consensus method can be considered useful for consulting Indigenous peoples about culturally appropriate best practice across different health services [30].

### Study aims

The Indigenous Health Research Priorities study involves community consultations in various regions of Queensland to identify health research priorities for mothers, fathers and babies, which will help to inform the future co-designed Indigenous Queensland Family Cohort Study [14]. The Health Research Priorities Study aims to engage with Indigenous communities in Queensland to identify health and medical research priorities for young families during preconception, pregnancy, post-partum, and early childhood. It seeks to determine these for Indigenous women and men and their young family members during the perinatal and early childhood period through a co-designed and collaborative process. The goal of the broader research study is to work in partnership using PAR approaches to co-identify health research priorities for families and communities and to the authors’ knowledge is the first study of its kind in Queensland. The objective is the successful prioritisation of the health research priorities identified with and for Indigenous Queensland families. This paper presents the study protocol, with preliminary data on participant demographics and feedback from participants about the acceptability of the research process and participating in the yarning sessions.

## Methods

### Study design

A qualitative research study design, using yarning methodology for focus groups or yarning sessions and workshops have and will continue to be undertaken in regions of Queensland, Australia. Yarning methodology involves “a semi-structured interview in an informal and relaxed discussion through which both the researcher and participant journey together visiting places and topics of interest relevant to the research study; yarning is a process that requires the researcher to develop and build a relationship that is accountable to Indigenous people participating in the research, and elicits participants’stories about their lived experiences, feelings, thoughts and ideas on the research topic’” [13], p. 38). Different types of yarns can be used in research, and encompass social, research and collaborative yarns [32].

The study will occur in three phases with Phase 1 being a series of yarning sessions, Phase 2 will involve the thematic analysis and development of research priority themes identified from the data and Phase 3 will be a Delphi workshop. This participatory action research (PAR) study was conceptualised in 2019 as a scoping exercise and research needs assessment for Indigenous communities in Queensland. The study entails a partnership approach for consultation, data collection, and confirmation of results with communities using a PAR approach.

### Consultation to establish the study and Indigenous leadership

**Fig. 1:**
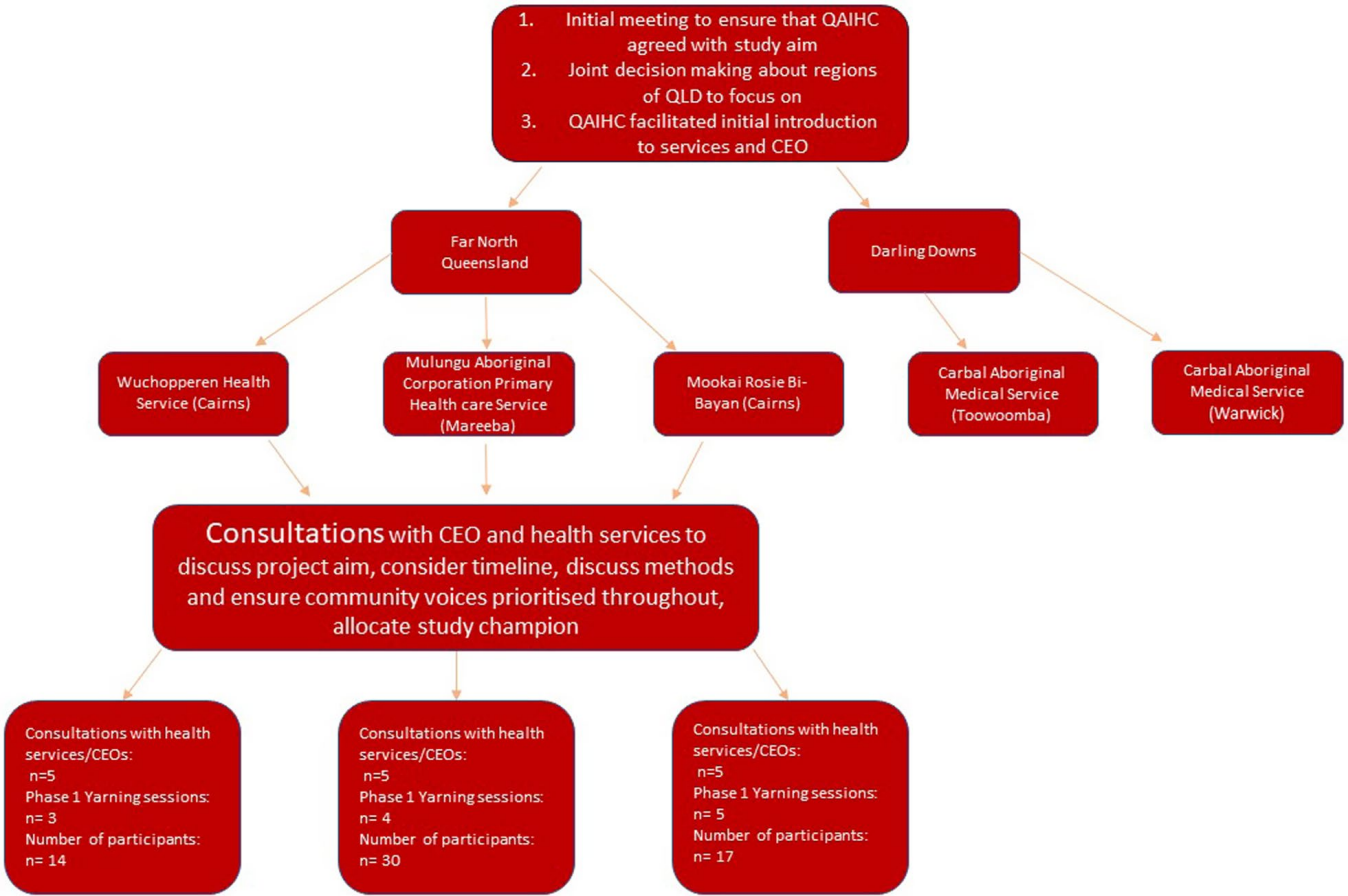
Diagram of consultations in Far North Queensland

Making time to consult with Indigenous communities has been embedded at all stages of this study. As a first step, preliminary face-to-face discussions were held with Queensland Aboriginal and Islander Health Council (QAIHC) to determine if their member services would find this proposed study beneficial. In the state of Queensland, QAIHC is the governing body for 31 community-controlled health services member organisations. With QAIHC’s support, services were identified, and an ongoing and regular series of meetings established to allow for rapport and trust building between the research team and the health services in the lead up to the study commencing (see Fig. 1).

In addition to seeking counsel from QAIHC, Indigenous leadership from two Senior Academics and Research Leaders has ensured that community engagement, the study protocol, data collection and future analysis meets Indigenous cultural protocols and community expectations.

All research team members have been involved in determining research aims, study design and data analysis. Research partnerships have been established with four community-controlled health services Chief Executive Officers and Deputies (to date), who have also been involved in study design, and have been integral to the planning and implementation of the study. Relationship building and on-going communication with community-controlled health services has been key to enable planning and processes to occur and to manage the challenges that have occurred as waves of the COVID-19 pandemic have been experienced.

### Study settings

**Fig. 2:**
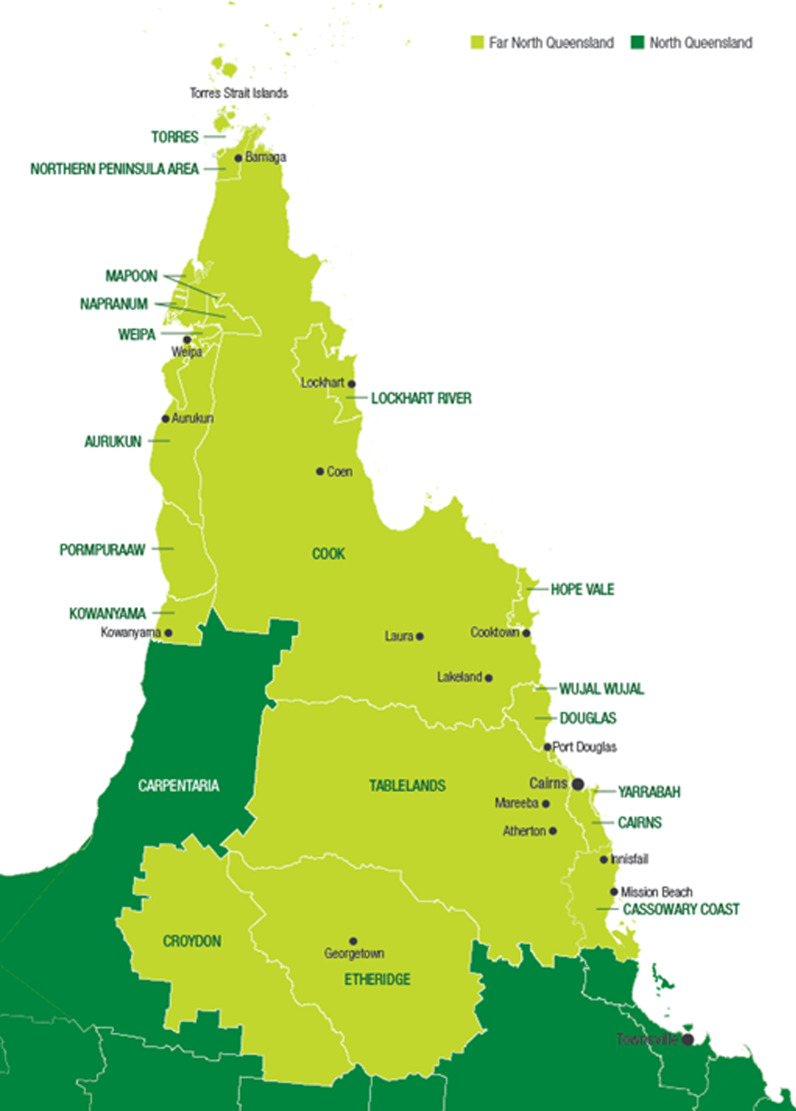
Map of North Queensland. *Source*: [49]

The study is being undertaken in the state of Queensland, Australia. This state has both Aboriginal and Torres Strait Islander populations and is the only Australian state that includes Torres Strait Islander territories. Three Aboriginal and Torres Strait Islander community-controlled health services in Far North Queensland are partnered as participant sites for the study: two in Cairns and one in Mareeba (see Fig. 2 Map of Far North Queensland). The partnering organisations are: Wuchopperen Health Service (Cairns), Mulungu Aboriginal Corporation Primary Health Care Service (Mareeba), and Mookai Rosie Bi-Bayan (Cairns). The fourth Aboriginal Community Controlled Health Services (ACCHS) is Carbal Aboriginal Medical Service in the South East area of Queensland, which is included as a site in this protocol, however data collection has not yet started at this location.

The Cairns area encompasses 1687 km^2^ of land along the coast between the Great Dividing Range and the Coral Sea. The region is a world-renowned tourist destination, and has World Heritage listed Wet Tropics rainforest to the west and north and the Coral Sea and World Heritage listed Great Barrier Reef Marine Park to the east [17]. Cairns has a population of 253,748 people counted in the 2021 Census with 10.6% identifying as Aboriginal and/or Torres Strait Islander [7]. In the Index of Relative Socio-economic Advantage and Disadvantage (IRSAD) many areas of Cairns are considered in the third quintile [4]. Wuchopperen is the largest community-controlled health service in Cairns and delivers culturally appropriate, comprehensive primary health care which includes medical and social and emotional wellbeing services for Indigenous peoples [55]. Wuchopperen offers continuity of care at the ante- and post-natal stages, including referrals and liaising with other health agencies and services around the Cairns region, and offers the Australian Nurse Family Partnership Program for first time mothers [55]. Wuchopperen has around 200 staff with approximately 60% identifying as Aboriginal and/or Torres Strait Islander.

Mookai Rosie Bi-bayan is a welcoming and culturally appropriate residential health care facility for women travelling from the Torres Strait Islands and Cape York Peninsula during pregnancy and for birthing their baby in Cairns. Women can stay during pre- and post-natal periods and can also be accommodated for other health care needs across the lifespan. Mookai Rose Bi-bayan can accommodate up to 24 clients, and is located in the outskirts of Cairns, providing a “home away from home… where women can feel safe and secure while they are spending those special days bonding with their new baby or recovering from medical treatment” [42]. Transport, meals, weekend outings for shopping and other activities such as fishing, arts, crafts, and attending cultural events are also offered. Mookai Rosie Bi-bayan is a community-controlled organisation, with a majority of Indigenous staff members including health professionals, catering staff, receptionists, program and administrative staff, and drivers. The Cape York Peninsula has a population of 7,803, with 47.7% female and 47.1% of the communities of these regions identifying as Aboriginal and/or Torres Strait Islander [3]. Due to its small population, and remoteness the ABS identifies Cape York in the IRSAD 1st quartile of social disadvantage [3].

Mareeba Shire is located on the Atherton Tablelands in Far North Queensland, 64 km southwest of Cairns, and has varied landscapes including World Heritage rainforest, waterfalls, agricultural farms, and cattle properties. The Shire’s estimated population is just over 22,000, with over 14% identifying as Indigenous compared with 4.6% in all of Queensland (ABS 2021 Census). Mareeba Shire is considered to be in the second most disadvantaged quintile in IRSAD [4]. Mulungu Aboriginal Primary Health Care Service (Mareeba) provides community-controlled and culturally appropriate primary health care across the life course, including clinical and social support to Indigenous families [43]. Mulungu has 80 staff with 95% identifying as Aboriginal and Torres Strait Islander [43].

A further site in the South-East sector of Queensland is Carbal Aboriginal Medical Service (AMS) with clinics in Toowoomba and Warwick, in the Darling Downs region. Toowoomba is a large regional centre, with a population of 162,059, with Aboriginal and/or Torres Strait Islander people making up 4.8% of the population [5]. Toowoomba and surrounding areas have a strong agricultural industry, and are considered in the third quintile in the IRSAD [4]. Warwick is a smaller regional town one hour south of Toowoomba, with a population of 12,294 in the 2021 Census, however the Aboriginal and/or Torres Strait Islander population is close to double that of Toowoomba, making up 8% of the population [6]. Carbal AMS was established in 2002 to improve access to a culturally appropriate and community-controlled responsive medical service across Toowoomba and Warwick, which offer parenting programs including “New Directions: Mums & Bubs program”, “Strong Mothers” and “Strong Fathers, Strong Families” programs [19].

Cairns’ region and Toowoomba have been chosen for their cultural and geographical diversity, and existing links with ACCHS in these two regions. As data is collected from these named sites the research team will be working towards establishing relationships with other organisations and undertaking research in other regions of Queensland. Sites that may be included in the future could be Mt Isa, Townsville, Rockhampton, Torres Strait Islands, Gold Coast and Brisbane.

### Researcher characteristics and reflexivity

The research was led by three women—one Aboriginal Research Officer (CN initially, then LW) and two non-Indigenous Researchers (KMR and LM). All researchers have experience conducting qualitative interviews with Indigenous people with over 15–20 years’ experience each in various research studies, and work closely together throughout this project in data collection, analysis, and writing. Having reflexivity in research approaches, enables flexible methods of generation, interpretation and analysis of data [16]. Indigenous research team members (LW, MT and BF) ensure the voices of all Indigenous community members are privileged, confirming the data analysis and reporting is trustworthy. Senior research team members have significant higher education, biomedical and research knowledge and include: one non-Indigenous Associate Professor (KMR) with experience conducting longitudinal cohort studies with Indigenous mothers and babies, and two Senior Indigenous Professors dedicated to Indigenous health and education programs and evaluations (MT and BF). The research team meet regularly to discuss the data and methods and reflect on researcher assumptions, with cultural mentorship provided by MT and BF.

Lead researcher (KMR) had established relationships with health service managers, which contributed to providing a welcoming environment to conduct the research. Both LM and LW have training, expertise and qualifications in both qualitative and Indigenous methods, which meant researchers were able to have more in-depth conversations with participants [32]. Author 1 (LM) has led and worked across seven qualitative Indigenous research studies through several universities and institutions since 2007. Author 2 (LW) is a Gamilaraay woman who is the Senior Aboriginal Research Officer and has worked in a longitudinal cohort study for Indigenous mothers and babies for 10 years and New South Wales and currently in Queensland, and ensures the approach taken has a cultural lens and centres Indigenous voices in the research processes.

### Participant characteristics

As this study is focused on families during the perinatal period it is important to include: (1) Indigenous community members, especially pregnant women, mothers, fathers, family members, kinship carers and Elders; and (2) health care professionals who work in maternal and infant, and early childhood health in roles that support families during this time of life. Each service identified that their health service staff were often Aboriginal and Torres Strait Islanders and/or they had significant experience in the areas of health research needs for their communities. We respectfully acknowledge that many Indigenous health professionals are also active community members and bring both perspectives to this study. With input from our partner ACCHS, we are including health workers from different areas of a health service who would like to participate in any aspect of the study, as we understand they are often community members, and may want to share their insight on health priorities in their communities.

### Recruitment and consent

Working with our partnered ACCHS, we identified a study champion at each service to assist planning of the study visits with the research team. The Participant Information and Consent Form (PICF) was sent to the study champion prior to yarning sessions to familiarise them with the study. All participants (health professionals and community members) received information about the study prior to attending on the day. Key health service contacts were encouraged to promote the yarning groups around their services via promotional flyers provided by the research team. Each site chose to hold separate yarning sessions for (i) health service staff and for (ii) community members. The study champion arranged the timing of yarning groups with staff members to ensure that key service contacts were available to attend. At some sites, yarning sessions were organised during staff meeting times, so it did not interrupt routine health service delivery. Community members were invited to participate by the health services, and this particularly included mothers and babies or family groups and Elders/Grandmothers groups.

At the start of the sessions, the research team spent time explaining the study, data management, and consent. Participants were informed that participation was voluntary, and that they could withdraw at any time without affecting their care, their relationship with staff, or the services they were accessing. The groups were advised that sessions would be audio recorded, and be de-identified at time of analysis, writing, and dissemination of results. Field notes would be taken by facilitators/researchers throughout the yarning session to aid in analysis.

It was also explained that future data could be kept by community/service as the data custodians/owners. Participants could select this option when completing their consent. Written informed consent was obtained prior to the start of the focus group session.

### Ethical considerations

This study was approved by a Hospital Human Research Ethics Committee (HREC/MML/72562 (V3)) and ratified by one university Human Research Ethics Committees (2022/HEO01885). Participants (including health service staff) were reimbursed with an AU$30 gift voucher from a department store for their time and contribution. A range of different incentive options had been discussed with partner services prior to study start and gift vouchers for a local department store was identified as being highly valued by participants.

### Data collection

Data will be collected during two different phases for this study (Phase 1 and 3).

#### Phase 1: Yarning sessions

Following signed consent to participate, participants complete a short demographic survey containing standard questions, such as age, number of children, education, employment status, income. This short survey had been piloted with our site champions at a prior visit to the health services and any changes suggested by key health care staff at this time were incorporated to ensure greater understanding of questions. These surveys are completed as participants settled in with a drink and snack and an informal, friendly conversation was held to make participants feel at ease. Research team members might discuss recent news, weather, or ask about local sites. Yarns were conducted face-to-face in a meeting or training room at health services.

The yarning sessions commenced with Aboriginal researcher (LW or CN) giving an Acknowledgement of Country, and then using a social yarning approach each researcher/facilitator undertook a brief self-introduction (LM, KMR). Each attending researcher spent time discussing their non-research background including; where they grew up, any connections to the community, their own families and why they are interested in this work. Not all research team members attended every session. This yarning approach was used to establish rapport and ensure participants felt comfortable to share their own stories [13]. Starting with a social yarn helped to initiate conversations, and encourage a more personal connection with participants to elicit open discussions [11, 32].

**Fig. 3:**
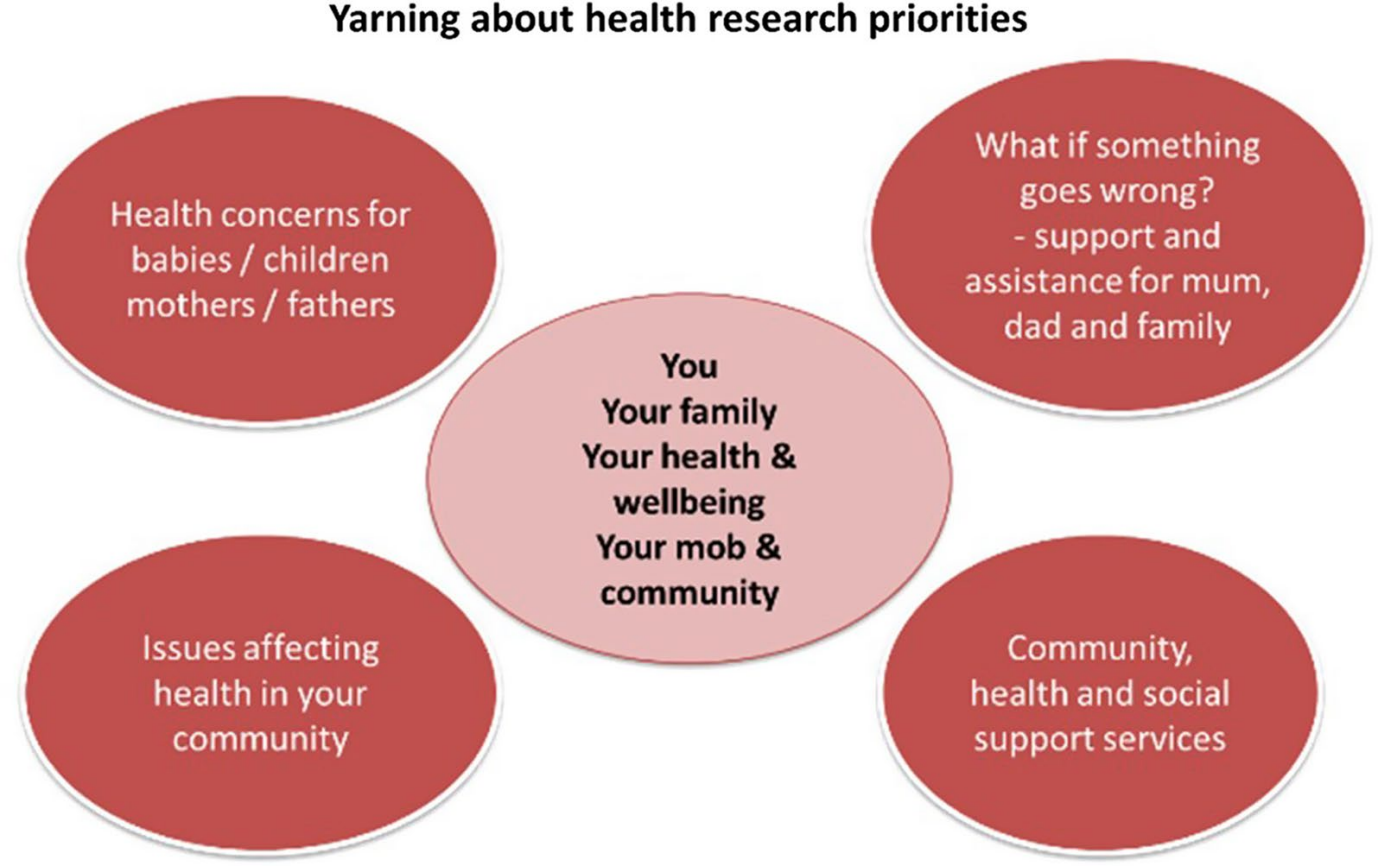
Yarning tool

Following introductions and this initial social yarn, the session started with a brief background and rationale for the study, leading the way for the research yarn [32]. To help orientate participants to the discussion and provide a visual aspect to the yarning topics, a yarning tool was used (see Fig. 3). Using a tool as a prompt for the yarns has been used effectively in other qualitative studies involving yarning sessions with women and community members [32, 38]. These tools were printed and distributed on tables and introduced the topics to be covered during the yarn, i.e., young family’s health needs, health issues affecting families/communities and services available. Researchers would highlight the yarning tool and give time and space for participants to start the yarn when ready, as they referred to the topics on the yarning tool and voluntarily started the topic yarns [32]. Alongside the yarning tool, a series of semi-structured questions were available for use by researchers as a prompt as needed to ensure the focus group stayed on track and all topics were covered. At times the yarns moved interchangeably between a research yarn, topic-focused yarn (addressing a specific topic from the yarning tool) and a more social yarn as participants became more comfortable sharing their stories [32].

To assess participant satisfaction with the research process, a feedback form was designed and collected at the end of the yarning sessions. This gauged participants’ satisfaction with taking part in a research focus group and asked about how they felt about what was covered during the yarning session. The feedback form constituted five open-ended questions, which asked about the best aspects of the yarning sessions, what feelings came to mind about taking part, suggested improvements, and other comments.

#### Phase 2: Identification of health research priorities list (analysis from phase 1)

Following transcription, audio recordings and transcripts are currently being reviewed by the research team to ensure accuracy. Two researchers (LM, LW*) including an Aboriginal researcher(*) conduct an initial thematic analysis, reading and then re-reading focus group transcripts, using open coding to identify broad themes directly from the data [33]. Main themes and subthemes are identified through inductive coding, discussed, refined, and confirmed with Senior Indigenous researchers (BF*, MT*) who read a selection of transcripts. NVivo 12 software (QSR International, Melbourne, 2012) was used to sort the data, and a coding tree was generated throughout the different stages of analysis. Data coding and analysis are conducted using a six-step process: (1) immersion in the data (self-transcription, repeated active reading/viewing); (2) generation of initial codes; (3) searching for themes; (4) reviewing themes; (5) defining and naming themes; and (6) producing the report including data extracts [16].

Harnessing the voices of women, families, and staff about health research priorities in communities was central to the thematic analysis of focus group transcripts. Thematic analysis generated a cross section of themes based on data collected during yarning sessions, with the aim being to identify health research priorities for young families in communities. However, there are other themes identified that fall outside of this aim. Health research topics which are beyond the scope of our study will be included in the prioritisation exercise, and a connection will be facilitated between participating health services and other research teams focusing on these research topics. As themes are extracted from the data an additional review will occur and a second review will be undertaken by all research team members collaboratively, which will form the basis for a list of health research priorities to be used in the Delphi study. The research team will discuss and consolidate the health research priorities identified and finalise these for Phase 3. An essential part of the preparation for Phase 3 will be to ensure that these have clear and simple names with an unambiguous definition attached.

#### Phase 3: Delphi study (to be conducted end of 2022 and through 2023)

During Phase 3, the research team will use a PAR approach that both returns the findings to communities, and allows community members to participate in a workshop to reach consensus on priorities through a Delphi study [31]. Three-hour workshops with health care staff and community members will be organised through the services. It is not necessary for the same participants who took part in the initial yarning sessions to attend these workshops. Where there are new participants joining the study for Phase 3, the recruitment and consent processes outlined in Phase 1 will be undertaken.

Workshops will begin by the research team presenting the identified themes from Phase 1 through digital or printed slides to the community and will ensure each participant has clear understanding of what each named theme means. Slides will show the overall themes, and then each overarching theme with corresponding sub-themes. The research team will aim to reach approximately 15–20 themes, with significant consensus about order of importance of these themes. The Dephi occurs in several ‘rounds’ and to encourage engagement and discussion with participants it will be undertaken around a series of tables with themes presented on cards similar to playing cards. Each individual participant will have a copy of every theme playing card in front of them. In Round 1 each participant will identify from their playing cards as either ‘yes’ important to me or ‘no’ not important to me on paper. The ‘yes’ votes for each theme will then be tallied up by researchers on a white board or butcher’s paper for all to view and the top two- thirds of themes will then be used again in Round 2. The bottom third of themes will be discarded with a researcher moving around the room and ensuring that each participant only has the agreed theme cards in front of them for the next round. The final score of each theme is the sum of scores of all participants. Delphi surveys are undertaken in multiple rounds and can have between four to six rounds or until sufficient consensus has been reached [18]. Due to time limitations, it is anticipated there will be at least two rounds conducted during the Phase 3 workshops.

Once data is obtained from all sites, the research priorities from each site will be considered to determine any differences between sites in each region, for example Far North Queensland and Darling Downs. It is envisaged that sites within regions may have the same research priorities, which would be beneficial for research ease. However, we acknowledge that each community has its own unique identity and may be likely to have areas of importance to their own community, which will require equal weighing in future research protocols.

### Data processing and storage

Yarning sessions are audio-recorded, and transcribed verbatim. Transcripts and audio files are de-identified and saved as password protected files on a shared drive only accessible to the research team. Transcripts are imported into NVivo 12 software for analysis (QSR International, Melbourne, 2012).

### Data ownership

Data, including surveys and voice recordings arising directly from participants remains the property of the participant however analysis from the data is owned by the research team. The data generated from the research study is be managed according to ethics approvals and the University’s Research Data Management Policy. This policy was developed to ensure that research data is properly managed according to recommendations made in The Australian Code for the Responsible Conduct of Research and applicable legislation.

### Preliminary findings

To date, qualitative data has been collected via yarning sessions and one-on-one interview with service providers, women, and families from May to August 2022. This paper is reporting preliminary data on participant demographics and feedback on the research process, from three services in Far North Queensland. Data analysis from yarning groups during Phase 1 is currently being undertaken and will be presented once completed, at a future date. On average the yarning sessions/interview ranged from 45 min to 1 h 50 min in length.

### Demographic survey

In total 61 people have taken part in either a focus group (*n* = 11) or individual interview (*n* = 1) at this time. These include community members who were health service clients, Elders, young mothers and other family members, and key health services staff involved in the delivery of pregnancy, maternal and infant health, early childhood and family wellbeing programs and services (see Table 1 for a breakdown of study participants). Most participants were < 50 years of age (53.3%), identified as Aboriginal (65%) and were female (93.4%). Fifty percent of participants had between three to five children, with almost 10% identifying that they cared for non-biological children.

**Table 1:** Study participants

Demographic characteristics (*n* = 61)	*n*	%
*Age range (years)*		
20–29 years	9	14.7
30–39 years	8	13.1
40–49 years	16	26
50–59 years	15	24.5
60–69 years	9	14.7
Missing	5	8.1
*Identity*		
Aboriginal	39	63.9
Torres Strait Islander	4	6.5
Aboriginal and Torres Strait Islander	8	13.1
Other	10	16.3
Missing	1	1.6
*Gender*		
Female	58	95
Male	4	6.5
Other/Prefer not to say	0	0
*Marital status*		
Single	25	40.9
Married	16	26.2
De facto	10	16.3
Separated	9	14.7
Missing	2	3.2
*Number of children*		
Nil	8	13.1
1 – 2	19	31.1
3 – 5	31	50.8
> 5	4	6.5
*Number of dependants who are not biological children*		
Nil	56	91.8
1–2	4	6.5
3–5	2	3.2
*Education*		
Less than high school	7	11.4
Completed high school	15	24.5
Some post-school education (TAFE, apprenticeship, university)	40	65.5
*Employment*		
Unemployed	6	9.8
Employed	46	75.4
Stay at home parent	9	14.7
Retired	1	1.6
*Role/s in community* ^*^(*n.b.* *n* = *9 respondents indicated they are a community member as well as their professional role*)		
Community member	21	34.4
Health care professional	18	29.5
Worker in organisations	25	40.9
Worker in policy and practice	2	3.2
Other	5	8.1
*Household income (annual)*		
$ 0	1	1.6
< $28,050	9	14.7
$28,051–$49,900	7	11.4
$49,901–$81,450	19	31.1
$81,451–$124,600	6	9.8
> = $124,601	2	3.2
Social assistance	1	1.6
Don’t know	4	6.5
Prefer not to say	6	9.8

### Yarning group feedback forms

Participant feedback responses were reviewed immediately following the yarning groups, to determine if the current research methods were considered suitable by the participants, and to gauge satisfaction with the research process. Word cloud was deemed a suitable mechanism to understand responses. Word cloud is a software tool that “digitally examines word frequencies. A word, concept, or term mentioned more often will be included in a larger font or text size in the word cloud and those mentioned less frequently will be included in a smaller font or won't be included at all. The visual representation or graphic portrays patterns of keywords and phrases included in the text, which allows viewers to identify relationships and meaning” [50], p. 51). Word clouds were generated from each of the open-ended questions on the feedback form using Nvivo 12 software (QSR Melbourne, 2021).

**Fig. 4:**
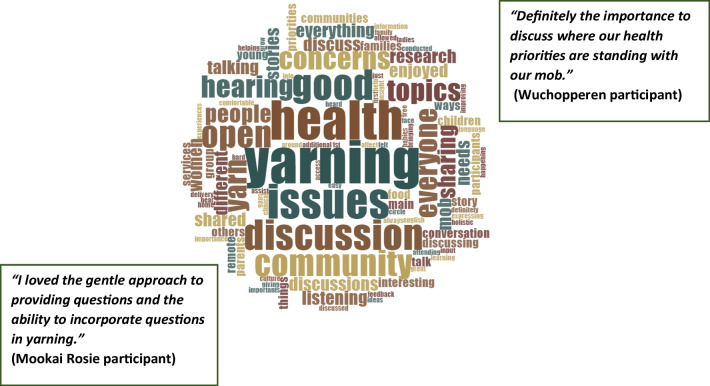
Word cloud based on answers to focus group feedback forms – Name the things you enjoyed the most about the focus group?

**Fig. 5:**
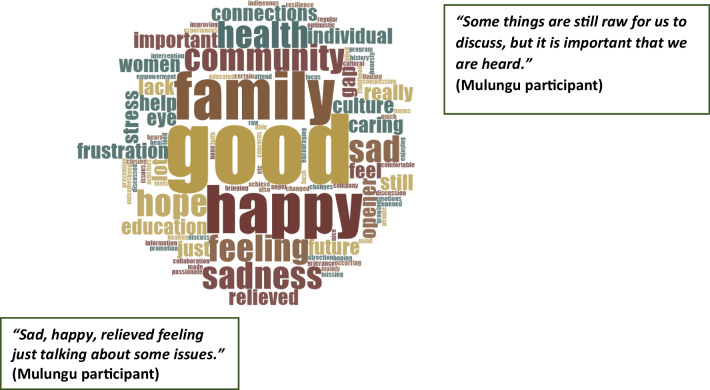
Wordcloud based on answers to focus group feedback forms – What words or feelings come to mind when you think about what we've talked about today? (one out of 56 people did not answer this question)

Of the 61 workshop participants, 55 answered and six did not complete the feedback form. Participants completed feedback forms while we completed paperwork related to gift cards. All participants were aware that they would receive a gift to thank them for their participation. When passing out the survey we encouraged them to be as honest as possible so that we could make sure our approaches were appropriate. Figure 4 highlights that when participants were asked what they enjoyed most about the yarning session, the predominant words used were ‘yarning, health, discussion, issues, community, open, hearing, mob and good’. Figure 5 depicts feedback to the question of what words or feelings come to mind about the discussions, with the most common answers being ‘happy, good, family, connections, caring, feeling and hope’. However, for some respondents, feelings such as ‘sadness, sad, stress’ resulting from the discussion were also noted.

## Discussion

Thematic analysis of transcripts from yarning sessions is still underway as is the continuation of this work to additional sites of Queensland. This project has undertaken and is continuing a systematic approach to the development of research priorities that is driven by Indigenous communities and will support the future of co-designed research. The high level of engagement in communities suggest that future work in these areas will have a likelihood of successful implementation across community and geographic contexts. The resultant health research priorities for Aboriginal and Torres Strait Islander communities generated by this study in Queensland study will be co-designed in strong partnered relationships with community organisations. Each community will receive a detailed and timely report compiled for their health services and regions which will present the health research priorities as highlighted, workshopped and decided on by their community members. While this report is ultimately about research it will likely facilitate the services’ strategic focus areas and future programs which are relevant to their services and communities.

The importance of listening and learning from Indigenous community members, the experts on their own health and wellbeing [28] was paramount in designing and conducting this research study. Following thematic analysis of Phase 1, the research team, and Indigenous Academic and Research leads (LW*, BF* and MT*) will distil these to research priority areas and revisit the same communities and conduct Phase 3, the Delphi part of the study, to prioritise the health research needs. This process ensures the importance of the voices of Indigenous communities in research is prioritised and guaranteed [45].

One of the key strengths of the study include the large engagement with services. This study is a testament to the strong partnerships established, an imperative for conducting co-design research approaches. The time taken to meet face-to-face and discuss the research study at length from the planning stages was beneficial in ensuring partner organisations were fully supportive and had multiple opportunities to discuss when, where why and how the research should be undertaken for their community and service. Minimal disruption to health service delivery and their staff role requirements was also guaranteed throughout the planning and implementation stages. Furthermore, this study is setting a foundation for future co-designed research with partnership organisations, with several community-controlled health services committing to supporting additional research being undertaken by this research team.

Another strength of the study includes providing the capacity to build skills and opportunities across partner organisations for increased participation in research. This has also been highlighted by a few studies implemented in ACCHS in the past [25, 28]. Study champions and key staff contacts have been identified in each participating service, who have taken part in the initial yarning sessions, and have shown an interest in continued involvement in the upcoming study phases. The opportunity of further upskilling in research through more involved participation in Phase 3 (the Delphi study) if time and work priorities permit, will be given to staff members.

Whilst the study includes the scope of perinatal period rather that all life stages and health areas, a further benefit envisaged is the opportunity to connect community-controlled services to other research groups if there is a health need identified by communities, that is beyond the scope of this perinatal research group. As mentioned, some participants highlighted in their feedback forms that considering and discussing other topics would be beneficial to communities. Exploring potential expansion in future to consider other life stages has been noted by the research team.

In most services, getting community members involved in the initial yarning sessions has been challenging to date, as reported through other research studies conducted in ACCHS [23, 32], however with most staff participants also identifying as members of the community this has been somewhat counteracted. Whilst we recognise that many participants are health workers and yarning sessions have been held mainly at health services, we also acknowledge that most health worker participants are also current community members and have shared their personal as well as professional experiences. Where possible, and at future sites we are aiming to target young women’s and young men’s groups, play groups and community centres to broaden the scope of the participants. The research team is continuing to liaise with maternal and infant health, and family wellbeing workers in the services to attend Mums and Bubs sessions, or playgroups, to facilitate and increase the chances of community members taking part in the research. Yarning sessions in person are the preferred method of data collection, however this can be challenging at times to fit around community priorities, and due to the continuing effects of the COVID pandemic, with reports of several community groups, programs and services not being reinstated since the earlier COVID lockdown phases.

## Conclusion

Closing the gap to end Indigenous health inequalities continues to be paramount, as it was since the start of this social justice initiative in 2007 (Australian Indigenous Health Infonet, 2022). This study aims to contribute to prioritising community ownership of research. It does this through the process of identifying health research priorities with, by and for communities, and reporting these back to health care services (not just ACCHS, but government health departments and non-government organisations) for planning future program, service, and research use. The study findings have the potential to outline health research needs more clearly for young families in Queensland Indigenous communities – to make a difference at a time when it counts the most.

## Data Availability

Not applicable.

## Abbreviations

ACCHS: Aboriginal Community Controlled Health Services
AMS: Aboriginal Medical Service
DOHaD: Developmental Origins of Health and Disease
IRSAD: Index of Relative Socio-economic Advantage and Disadvantage
PAR: Participatory Action Research
PICF: Participant Information and Consent Form
QAIHC: Queensland Aboriginal and Islander Health Council
